# Size-Specific Particulate Matter Associated With Acute Lower Respiratory Infection Outpatient Visits in Children: A Counterfactual Analysis in Guangzhou, China

**DOI:** 10.3389/fpubh.2021.789542

**Published:** 2021-12-02

**Authors:** Zhenyu Liang, Qiong Meng, Qiaohuan Yang, Na Chen, Chuming You

**Affiliations:** Department of Pediatrics, Guangdong Second Provincial General Hospital, Guangzhou, China

**Keywords:** particulate matter, lower respiratory infection, particle, China, children

## Abstract

The burden of lower respiratory infections is primarily evident in the developing countries. However, the association between size-specific particulate matter and acute lower respiratory infection (ALRI) outpatient visits in the developing countries has been less studied. We obtained data on ALRI outpatient visits (*N* = 105,639) from a tertiary hospital in Guangzhou, China between 2013 and 2019. Over-dispersed generalized additive Poisson models were employed to evaluate the excess risk (ER) associated with the size-specific particulate matter, such as inhalable particulate matter (PM_10_), coarse particulate matter (PM_c_), and fine particulate matter (PM_2.5_). Counterfactual analyses were used to examine the potential percent reduction of ALRI outpatient visits if the levels of air pollution recommended by the WHO were followed. There were 35,310 pneumonia, 68,218 bronchiolitis, and 2,111 asthma outpatient visits included. Each 10 μg/m^3^ increase of 3-day moving averages of particulate matter was associated with a significant ER (95% CI) of outpatient visits of pneumonia (PM_2.5_: 3.71% [2.91, 4.52%]; PM_c_: 9.19% [6.94, 11.49%]; PM_10_: 4.36% [3.21, 5.52%]), bronchiolitis (PM_2.5_: 3.21% [2.49, 3.93%]; PM_c_: 9.13% [7.09, 11.21%]; PM_10_: 3.12% [2.10, 4.15%]), and asthma (PM_2.5_: 3.45% [1.18, 5.78%]; PM_c_: 11.69% [4.45, 19.43%]; PM_10_: 3.33% [0.26, 6.49%]). The association between particulate matter and pneumonia outpatient visits was more evident in men patients and in the cold seasons. Counterfactual analyses showed that PM_2.5_ was associated with a larger potential decline of ALRI outpatient visits compared with PM_c_ and PM_10_ (pneumonia: 11.07%, 95% CI: [7.99, 14.30%]; bronchiolitis: 6.30% [4.17, 8.53%]; asthma: 8.14% [2.65, 14.33%]) if the air pollutants were diminished to the level of the reference guidelines. In conclusion, short-term exposures to PM_2.5_, PM_c_, and PM_10_ are associated with ALRI outpatient visits, and PM_2.5_ is associated with the highest potential decline in outpatient visits if it could be reduced to the levels recommended by the WHO.

## Introduction

Lower respiratory infections, such as pneumonia and bronchiolitis, are the sixth leading cause of death in all age groups, resulting in ~2.4 million deaths worldwide in 2016 (1). The burden of lower respiratory infections is unevenly distributed across the world and is primarily born in the developing countries with socioeconomically disadvantaged communities, where proper nutrition, clean fuel, sanitation, and clean air are unavailable or inadequate (2, 3). China has experienced a staggering economic growth in the past 30 years, resulting in a steady increase in the life expectancy and improvement in the health outcomes in the country. From 1990 to 2019, the number of cases and mortalities of lower respiratory infections declined by 21.98 and 65.94%, respectively. In 2019, there were still 55.84 million cases, with 185,264.33 mortalities attributed to lower respiratory infections in China, making it the country's leading cause of mortality in children under-five (4).

Exposure to ambient particulate matter has been widely reported to be associated with lower respiratory infections (5–7). However, evidence on the association between size-specific particulate matter and lower respiratory infections, especially that from the developing countries where the level of air pollution is high, is relatively limited (8–10). Based on particle diameter, inhalable particulate matter (PM_10_) can be divided into fine particles (PM_2.5_) and coarse particles (PM_c_). Most studies only focused on the health effects of PM_2.5_, while the effects of PM_10_ and PM_c_ remain inconclusive.

The previous time-series studies that examined the association between the size-specific particulate matter and the risk of adverse health outcomes often reported the odds ratio or excess risk (ER) estimates per 1 or 10 μg/m^3^ (11– 13). However, these effect size estimates ignore the underlying statistical distributions of the air pollutants and may not be comparable across the size-specific particulate matter. In this study, we introduced the counterfactual analyses to effectively compare the potential reduction of acute lower respiratory infection (ALRI) hospitalizations (counterfactual outcomes) associated with the size-specific particulate matter (14). These counterfactual outcomes, which accounted for the statistical distributions of air pollutants, can be directly comparable for different particulate matter and, therefore, have more public health implications for policymakers.

In this current study, we investigate the association between the size-specific particulate matter and the outpatient visits of ALRI. Beyond these analyses, we further employed a counterfactual approach to investigate the potential percent reduction of ALRI outpatient visits if the levels of particulate matter were as low as those recommended by the WHO.

## Methods

### Acute Lower Respiratory Infection Data

**Figure 1:**
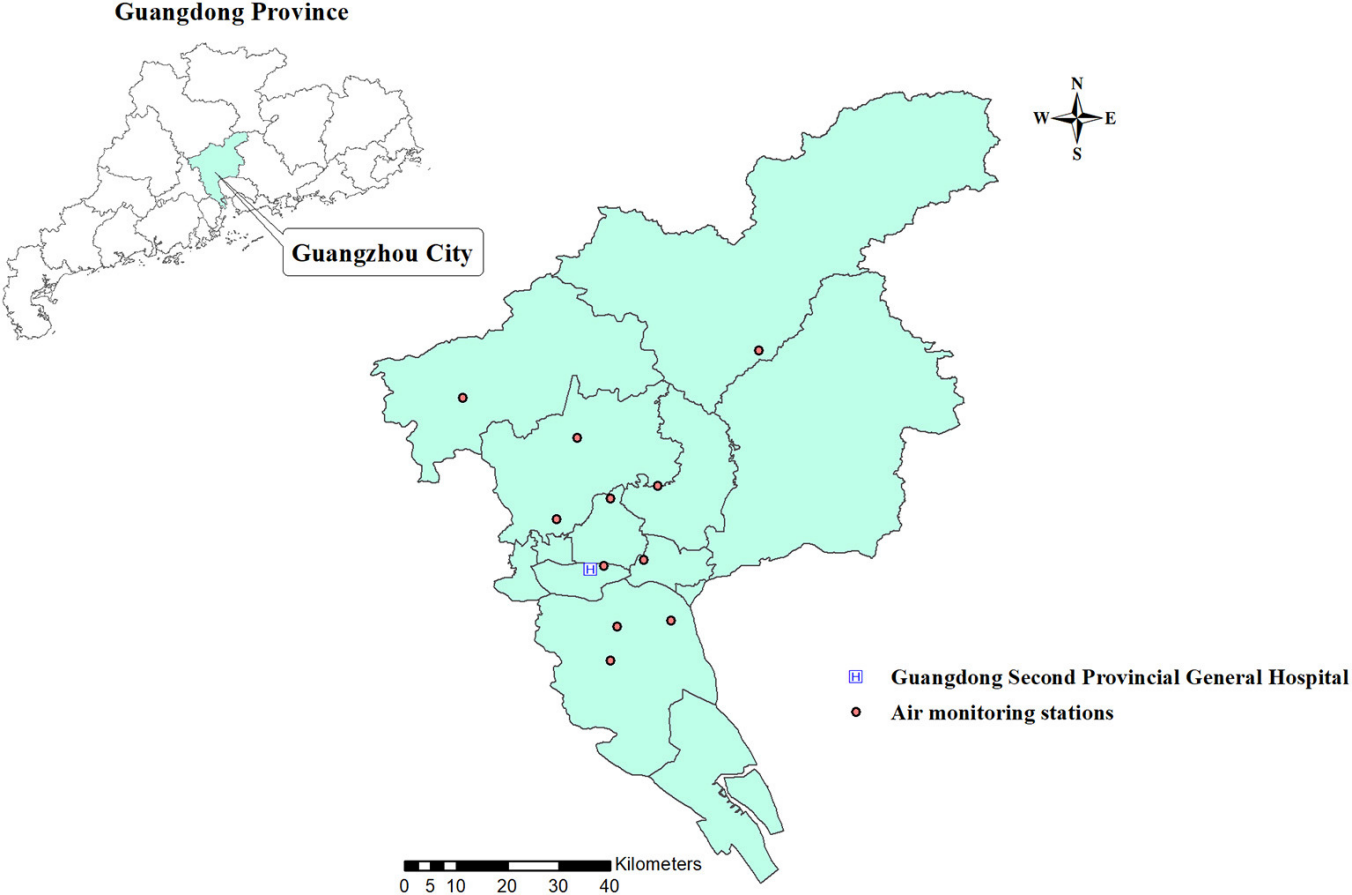
Geographical distribution of the sample hospitals and air monitoring stations.

This study is a time-series analysis of ALRI outpatient visits from 2013 to 2019 in Guangzhou, China. Data on ALRI-related hospital outpatient visits were retrieved from the Guangdong Second Provincial General Hospital, which is located in the southwest of the city (Figure 1). This is one of the tertiary hospital in Guangzhou (15). According to the International Classification of Diseases, Tenth Revision (ICD-10), hospital outpatient visits with the primary diagnoses of pneumonia (J12-J18), bronchiolitis (J20-J21), and asthma (J45-J46) (16) were obtained between February 2013 and December 2019. We aggregated the three subtypes of ALRI into a series of daily time-series data (17–20).

### Air Pollution and Meteorological Data

Daily concentrations of air pollution during the study period were obtained from 11 air monitoring stations in Guangzhou (Figure 1), such as PM_10_, PM_c_, PM_2.5_, nitrogen dioxide (NO_2_), sulfur dioxide (SO_2_), and ozone (O_3_). Following a previous study (19), the PM_c_ concentrations were calculated by the subtracting PM_2.5_ from PM_10_, because PM_10_ consists of PM_2.5_ and PM_c_. Details on the measurement of air pollutants have been described previously (21). Approximately 1% of observation days had missing data for air pollutants, and a linear interpolation approach was used to fill in the missing data (the “na.approx” function in “zoo” package in R).

Daily meteorological data (mean temperature and relative humidity [RH]) were obtained from the National Weather Data Sharing System (http://data.cma.cn/). Because there is a potentially high correlation between different air pollutants and meteorological factors, we examined the Pearson correlation coefficients among these variables (22, 23).

### Statistical Models

The ALRI data, daily air pollution concentrations, and meteorological data were linked by date. Following similar epidemiologic studies (11, 12), the association between particulate matter and hospital outpatient visits for ALRI was examined using a generalized additive over-dispersed Poisson model (GAM), where the property of over dispersion was tested using the approach proposed by Cameron and Trivedi (24) (Supplementary Table 1). In the model, public holidays (PH) and days of the week (DOW) were adjusted as categorical variables. Seasonal patterns, long-term trends, temperature, and RH were controlled through smoothing splines. Following the approaches used in the previous studies (25, 26), we selected six degrees of freedom (df) per year for temporal trends, a df of six for moving average temperature of the current day and the previous 3 days (Temp03), and RH.

Considering the delayed health effects of air pollutants, we examined the lag effects for different lag structures. We began with the same day (lag0) up to a 5-day lag (lag5) in the single-lag day models. We also considered the accumulated effects of multi-day lags (moving averages for the current day and the previous 1, 2, and 3 days [lag01, lag02, and lag03]).

### Stratified Analyses

To evaluate the potential effect modifiers of the particulate matter–ALRI associations, we conducted the stratified analyses by sex (men vs. women), age group (age <5 vs. age 5–14), and season (warm *vs* cold). The warm season was defined as the period from April to September, and the cold season was from October to March. The 95% CI of the difference between the groups was calculated using the following formula:


$$Q_{1}-Q_{2}\;\pm \;1.96\sqrt{{\left(SE_{1}\right)}^{2}+{\left(SE_{2}\right)}^{2}}$$

where Q represents the estimated coefficient in each stratum, and SE is the corresponding standard error (27). The difference was considered statistically significant if the 95% CI did not include unity.

### Counterfactual Analyses on the Burden of ALRI Attributable to Air Pollution

We estimated the burden of ALRI attributable to PM_2.5_, PM_c_, and PM_10_ by calculating the difference between the observed ALRI outpatient visits and the counterfactual visits predicted using well-recognized reference values of particulate matter recommended by the WHO (28) and our previously built generalized additive over-dispersed Poisson models. This difference between the observed and counterfactual ALRI outpatient visits represents the estimated burden of ALRI outpatient visits associated with the size-specific particulate matter. The counterfactual scenarios were set to be hypothetical values of PM_2.5_ and PM_10_ set by the recently updated WHO Global Air Quality Guidelines (24 h mean: 15 μg/m^3^ for PM_2.5_ and 45 μg/m^3^ for PM_10_) (28). However, PM_c_ was not directly regulated by the WHO Air Quality Guidelines, the reference concentration for PM_c_ (30 μg/m^3^) was defined as the difference between the standard concentrations of PM_10_ and PM_2.5_ according to the previous studies (17, 20). The observed air pollution levels lower than the reference values were kept the same in the counterfactual scenario. The 95% CIs were constructed using 1,000 bootstrap replicates with a replacement for each model (29, 30).

### Sensitivity Analyses

We applied a series of sensitivity studies to examine the accuracy of the main models. The main findings were assessed by changing the df in the smooth functions for temporal trends and meteorological factors. Additionally, we adjusted for the gaseous air pollutants (SO_2_, NO_2_, and O_3_) in two-pollutant models. The models were regarded as robust if there were no significant changes after df-change or further adjustment for gaseous air pollutants.

In all statistical analyses, a *p* ≤ 0.05 was considered statistically significant. All data cleaning, aggregation, and visualization, and statistical analyses were performed using the statistical computing environment R version 4.0.5 (31).

## Results

Figure 1 presents the geographical location of Guangzhou and the sample hospital, as well as the geographical distribution of the air monitoring stations in Guangzhou. A total of 105,639 pediatric outpatient visits were included in the study, with the following breakdown of cases: 35,310 pneumonia, 68,218 bronchiolitis, and 2,111 asthma. Table 1 shows the summary statistics of ALRI subtypes, size-specific particulate matter (PM_10_, PM_c_, and PM_2.5_), and gaseous pollutants (SO_2_, NO_2_, and O_3_). The daily averages (SD) of pneumonia, bronchiolitis, and asthma cases were 12.5 (9.1), 24.3 (11.5), and 0.8 (1.4), respectively. The mean concentrations of PM_10_, PM_c_, and PM_2.5_ in our study were 58.3, 21.0, and 37.8 μg/m^3^. The mean (SD) of temperature and relative humidity was 22.8°C (5.9) and 81.8% (10.2%), respectively.

**Table 1:** Summary statistics of acute lower respiratory infections outpatient visits, air pollutants, and meteorological variables.

	**Mean**	**SD**	**Percentile**				
			**Min**	**25th**	**50th**	**75th**	**Max**
**Acute lower respiratory infections**							
Pneumonia (*N* = 35,310)	12.5	9.1	0.0	6.0	11.0	16.0	73.0
Bronchiolitis (*N* = 68,218)	24.3	11.5	0.0	16.0	23.0	31.0	81.0
Asthma (*N* = 2,111)	0.8	1.4	0.0	0.0	0.0	1.0	12.0
**Air pollution, μg/m** ^**3**^							
PM_10_	58.3	28.1	10.0	38.2	51.1	73.4	217.8
PM_c_	21.0	9.9	0.8	14.7	18.8	25.3	77.7
PM_2.5_	37.8	21.2	4.6	22.7	32.3	48.3	156.4
SO_2_	13.6	8.5	2.8	8.6	11.9	16.5	166.4
NO_2_	45.2	18.6	4.4	33.6	41.2	53.7	177.7
O_3_	51.6	30.2	3.5	30.0	47.2	67.1	294.6
**Meteorological variables**							
Temperature, °C	22.8	5.9	1.7	19.0	25.0	27.5	32.8
Relative humidity, %	81.8	10.2	30.5	77.0	83.1	89.3	100.0

*SD, standard deviation*.

**Figure 2:**
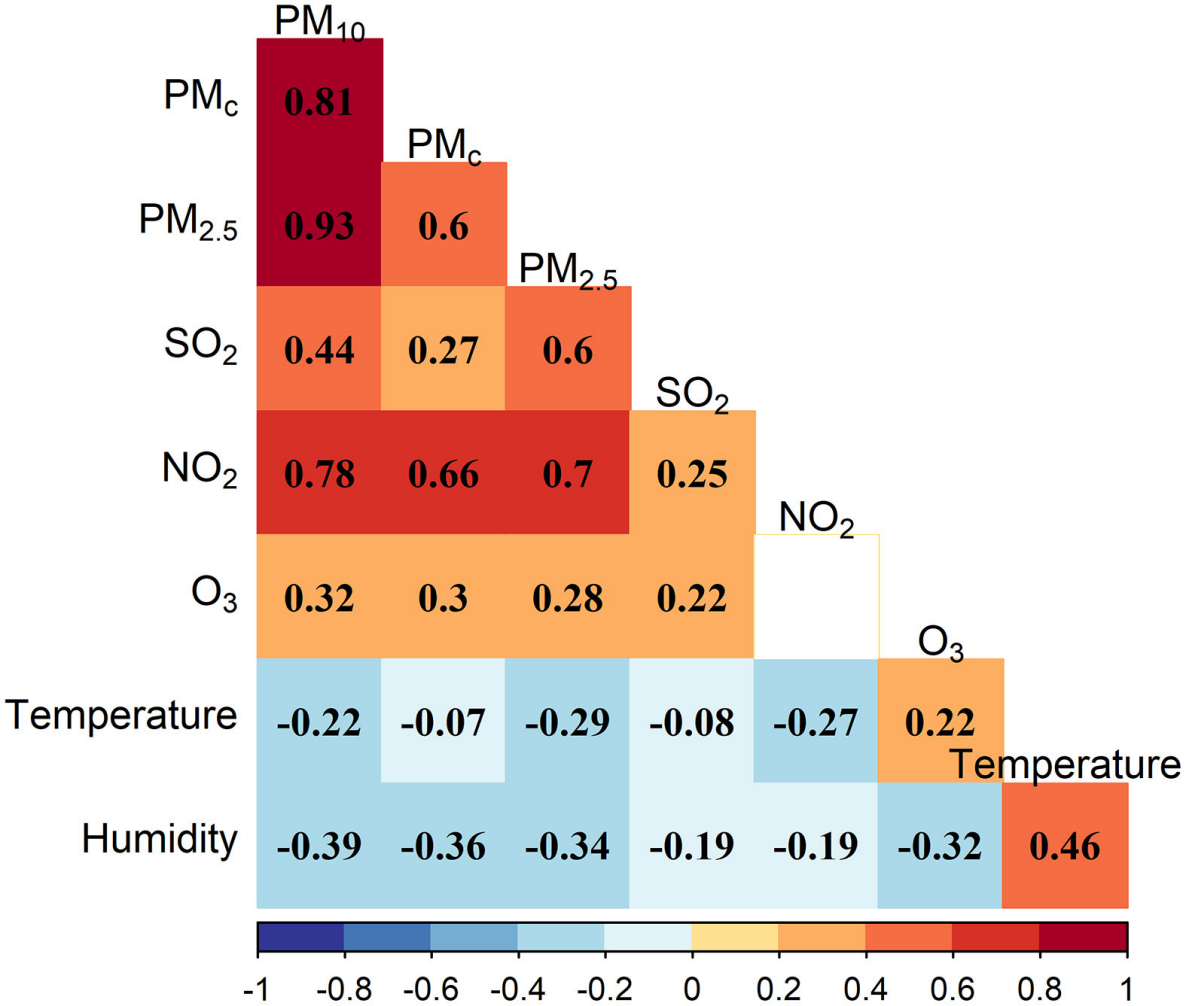
Correlation plot of the air pollutants and meteorological variables.

Figure 2 shows the correlation plot of the air pollutants and meteorological variables in our sample. All the Pearson's correlation coefficients were statistically significant except for the correlation between NO_2_ and O_3_. PM_10_ was significantly and strongly correlated with PM_c_ and PM_2.5_ (Pearson's correlation coefficients: 0.81 and 0.93); NO_2_ was moderately correlated with particulate matters (Pearson's correlation coefficients for PM_10_, PM_c_, and PM_2.5_: 0.78, 0.66, and 0.70). Meteorological variables were negatively correlated with air pollutants except for the positive correlation between temperature and O_3_.

Table 2 exhibits the ER of pneumonia, bronchiolitis, and asthma outpatient visits associated with per 10 μg/m^3^ increase in PM_2.5_, PM_c_, and PM_10_ at lag03. The results revealed that size-specific particulate matter were significantly associated with pneumonia, bronchiolitis, and asthma, respectively, in single-pollutant models, where the ER of PM_c_ was the largest, followed by that of PM_2.5_ and PM_10_. The results were consistent and robust in two-pollutant models with further adjustment for SO_2_, NO_2_, and O_3_, except for those asthma models controlling for NO_2_. The corresponding exposure–response non-linear curves for the daily particulate matter and log relative risk are shown in Supplementary Figure 1.

**Table 2:** Excess risk and 95% CIs of pneumonia, bronchiolitis, and asthma for each 10 μg/m^3^ increase in PM_2.5_, PM_c_, and PM_10_ using single- and two-pollutants models at lag03.

**Pollutants**	**Models**	**Pneumonia**	**Bronchiolitis**	**Asthma**
**PM**_**10**_				
	Single-pollutant model	**3.71 (2.91, 4.52)**	**3.21 (2.49, 3.93)**	**3.45 (1.18, 5.78)**
	Two-pollutant models			
	Control for SO_2_	**3.81 (2.97, 4.66)**	**3.44 (2.69, 4.21)**	**3.46 (1.13, 5.85)**
	Control for NO_2_	**2.47 (1.47, 3.47)**	**1.48 (0.58, 2.37)**	0.26 (−2.58, 3.19)
	Control for O_3_	**4.06 (3.22, 4.91)**	**3.48 (2.72, 4.25)**	**3.72 (1.30, 6.20)**
**PM**_**c**_				
	Single-pollutant model	**9.19 (6.94, 11.49)**	**9.13 (7.09, 11.21)**	**11.69 (4.45, 19.43)**
	Two-pollutant models			
	Control for SO_2_	**9.32 (6.98, 11.72)**	**9.72 (7.58, 11.91)**	**11.70 (4.29, 19.63)**
	Control for NO_2_	**5.58 (3.03, 8.19)**	**4.80 (2.45, 7.20)**	3.26 (−4.88, 12.09)
	Control for O_3_	**9.52 (7.21, 11.87)**	**9.47 (7.37, 11.61)**	**12.09 (4.56, 20.17)**
**PM**_**2.5**_				
	Single-pollutant model	**4.36 (3.21, 5.52)**	**3.12 (2.10, 4.15)**	**3.33 (0.26, 6.49)**
	Two-pollutant models			
	Control for SO_2_	**4.61 (3.37, 5.87)**	**3.50 (2.39, 4.63)**	**3.45 (0.14, 6.87)**
	Control for NO_2_	**2.30 (0.96, 3.65)**	0.39 (−0.78, 1.58)	−0.40 (−3.86, 3.18)
	Control for O_3_	**4.85 (3.63, 6.09)**	**3.38 (2.29, 4.48)**	**3.54 (0.26, 6.91)**

*The bold type represents the statistically significant (p < 0.05). The underline indicates the two-pollutant models are models that use PM_2.5_, PM_c_, or PM_10_ as the main air pollutant, with further adjustment for one of the gaseous pollutants (SO_2_, NO_2_, O_3_)*.

**Figure 3:**
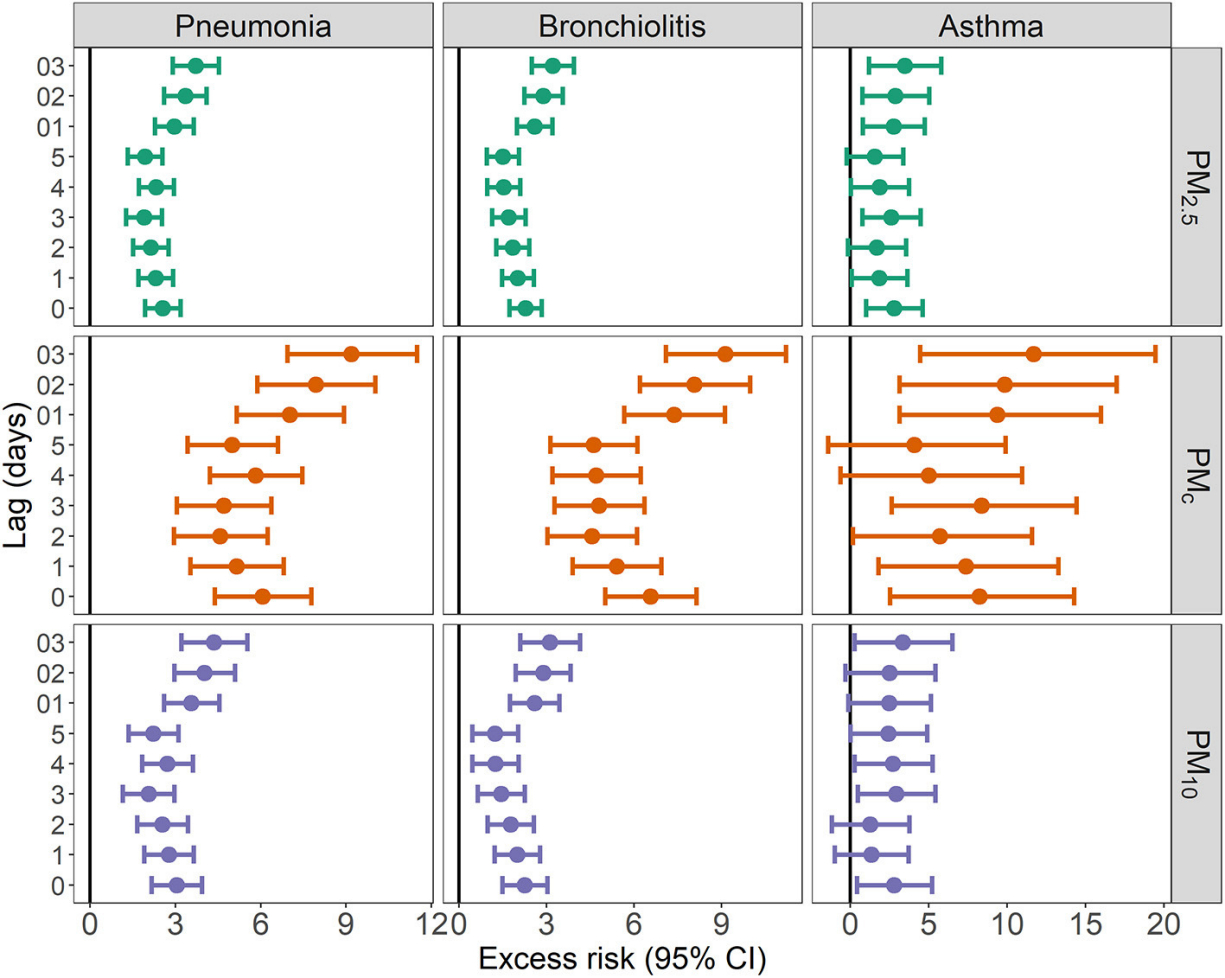
Excess risk (95% CIs) of hospital outpatient visits associated with 10 μg/m^3^ increase in PM_10_, PM_c_, and PM_2.5_.

Similar patterns of ER of ALRI outpatient visits associated with per 10 μg/m^3^ increase in the size-specific particulate matter could be observed in Figure 3. Each 10 μg/m^3^ increase in PM_2.5_, PM_c_, and PM_10_ was associated with the outpatient visits for pneumonia, bronchiolitis, and asthma on different lag days. In contrast, the effects of the size-specific particulate matter on asthma are less robust: the moving average lags of PM_c_, and PM_2.5_ were still significantly associated with the ALRI outpatient visits, but the effects of lag0 to lag5 of PM_c_ and PM_2.5_ and different lags of PM_10_ were non-significant or at borderline significant. Sensitivity analyses using the different degrees of freedom for splines of temporal trends and temperature showed a generally consistent pattern (Supplementary Table 2).

Table 3 presents the estimated ER with 95% CI of pneumonia, bronchiolitis, and asthma stratified by sex, age group, and season, where the bold numbers indicate the significant differences across strata. We observed that each 10 μg/m^3^ increase in PM_10_, PM_c_, and PM_2.5_ was consistently associated with significantly different effects on pneumonia outpatient visits by sex and season groups. Similar differential effects were observed for the bronchiolitis associated with increases in PM_10_ and PM_c_ by different season strata, but not for PM_2.5_. However, the differential effects across strata were much less significant for the asthma outpatient visits: it was only significantly different between the warm and cold seasons.

**Table 3:** Excess risk and 95% CIs of pneumonia, bronchiolitis, and asthma for each 10 μg/m^3^ increase in PM_2.5_, PM_c_, and PM_10_ stratified by gender, age group, and season.

**Pollutants**	**Stratum**	**Pneumonia**	**Bronchiolitis**	**Asthma**
**PM**_**10**_				
	**Gender**			
	Male	**4.49 (3.54, 5.45)**	3.44 (2.68, 4.21)	4.46 (1.62, 7.39)
	Female	**2.68 (1.61, 3.75)**	2.76 (1.84, 3.69)	1.78 (−1.51, 5.18)
	**Age**			
	<5	3.50 (2.66, 4.34)	3.09 (2.36, 3.82)	1.78 (−0.97, 4.60)
	5–14	4.50 (2.71, 6.33)	3.70 (2.49, 4.92)	6.01 (2.39, 9.75)
	**Season**			
	Warm	–**0.06 (**–**1.24, 1.13)**	**2.13 (1.03, 3.23)**	6.53 (2.52, 10.69)
	Cold	**5.12 (4.00, 6.25)**	**3.76 (2.74, 4.79)**	1.76 (−1.00, 4.61)
**PM**_**c**_				
	**Gender**			
	Male	**11.07 (8.36, 13.83)**	10.02 (7.82, 12.25)	15.65 (6.36, 25.74)
	Female	**6.70 (3.78, 9.71)**	7.57 (4.99, 10.21)	5.94 (-3.96, 16.87)
	**Age**			
	<5	8.69 (6.37, 11.06)	8.76 (6.69, 10.88)	6.96 (−1.69, 16.38)
	5–14	10.76 (5.65, 16.13)	10.75 (7.34, 14.27)	17.98 (6.46, 30.74)
	**Season**			
	Warm	–**0.93 (**–**4.33, 2.60)**	**3.77 (0.40, 7.26)**	22.14 (6.92, 39.53)
	Cold	**13.57 (10.46, 16.77)**	**11.92 (9.07, 14.84)**	9.39 (0.93, 18.56)
**PM**_**2.5**_				
	**Gender**			
	Male	**5.31 (3.95, 6.69)**	3.48 (2.39, 4.58)	4.13 (0.30, 8.09)
	Female	**3.11 (1.58, 4.65)**	2.45 (1.14, 3.78)	1.92 (-2.54, 6.58)
	**Age**			
	<5	4.07 (2.87, 5.28)	2.88 (1.84, 3.93)	1.53 (−2.16, 5.35)
	5–14	5.59 (3.07, 8.17)	4.10 (2.39, 5.85)	6.13 (1.22, 11.28)
	**Season**			
	Warm	**0.08 (**–**1.49, 1.67)**	3.00 (1.56, 4.46)	**7.73 (2.62, 13.08)**
	Cold	**6.08 (4.42, 7.77)**	3.01 (1.51, 4.53)	–**0.30 (**–**4.05, 3.60)**

*The bold type represents the statistically significant differences (p < 0.05)*.

*Warm season: April to September; cold season: October to March*.

Table 4 shows the proportion reduction of ALRI (pneumonia, bronchiolitis, and asthma) outpatient visits attributable to PM_2.5_, PM_c_, and PM_10_ in Guangzhou from 2013 to 2019 using a counterfactual analysis framework (15 μg/m^3^ for PM_2.5_, 30 μg/m^3^ for PM_c_, and 45 μg/m^3^ for PM_10_). We found that PM_2.5_ was associated with the largest decline in ALRI outpatient visits (pneumonia: 11.07%, 95% CI: [7.99, 14.30%]; bronchiolitis: 6.30% [4.17, 8.53%]; asthma: 8.14% [2.65, 14.33%]) if the levels of air pollution were reduced to the level of the reference guidelines.

**Table 4:** Counterfactual analysis on the percent of decline (95% confidence intervals) in acute lower respiratory infection outpatient visits if the level of PM_2.5_, PM_c_, and PM_10_ were reduced to the reference levels in Guangzhou from 2013 to 2019.

	**Pneumonia**	**Bronchiolitis**	**Asthma**
PM_10_	**7.54% (5.80, 9.35%)**	**5.98% (4.57, 7.44%)**	**6.34% (0.47, 13.05%)**
PM_c_	**1.33% (0.99, 1.67%)**	**1.46% (1.13, 1.81%)**	**1.78% (0.64, 3.07%)**
PM_2.5_	**11.07% (7.99, 14.30%)**	**6.30% (4.17, 8.53%)**	**8.14% (2.65, 14.33%)**

*The references of PM_10_, PM_2.5_, and PM_c_ concentration were 45 μg/m^3^ for PM_10_, 30 μg/m^3^ for PM_c_, and 15 μg/m^3^ for PM_2.5_, respectively*.

*The bold type represents the statistically significant (p < 0.05)*.

## Discussion

In this study, we observed statistically significant ERs and a potential decline of ALRI (such as pneumonia, bronchiolitis, and asthma) outpatient visits associated with the size-specific particulate matter. The results were consistent in exposure assessment using different lags (lag 0–5 and moving averages of 1–3 days), two-pollutant models adjusting for SO_2_, NO_2_, and O_3_, and various degrees of freedom. In counterfactual analyses that are of more public health significance, PM_2.5_ was associated with the largest decline in the ALRI outpatient visits if the exposure was as low as the WHO reference guideline.

Consistent with a previous study (18), we observed dissimilar effect estimates associated with the size-specific particulate matter, and the largest ER was found to be that of PM_c_, followed by that of PM_2.5_ and PM_10_. However, these results should be interpreted with caution as PM_2.5_, PM_c_, and PM_10_ have different means and standard deviations: the mean of PM_c_ in our sample (21.0 μg/m^3^) was lower than the reference level (30 μg/m^3^); the SD of PM_c_ (9.9 μg/m^3^) was much smaller compared with that of PM_2.5_ (21.2 μg/m^3^) and PM_10_ (28.1 μg/m^3^). Therefore, the ER of ALRI associated with PM_c_ appeared to be the largest, which likely resulted from its smaller SD.

We found a larger effect of particulate matter–ALRI association among men than women, which is similar to the results of the sex-specific effects of particulate matter pollution reported previously (19). This may be due to the biological differences between men and women populations, such as hormones, sizes of airway diameters and lung sizes, and build, which will, in turn, result in the difference in the transport of pollutants and tissue deposition (17, 32). In addition, the observed associations between particulate matter and ALRI were stronger during the cold season, which is in line with the several previous studies (26, 33–35). There are several possible biological mechanisms, such as season-specific behavior, differences in PM_2.5_ levels, constituent, and etiologic agents, which may be responsible for this seasonal difference.

The effects of particulate matter pollution on ALRI did not seem to be confounded by SO_2_ and O_3_. However, the associations between particulate matter pollution and ALRI decreased after adjusting for NO_2_, in particular, the particulate matter-asthma associations became non-significant. It was difficult to ascertain their potential effects especially given the potential multicollinearity issue, possibly because NO_2_ was highly correlated with particulate matter (Figure 2).

Given the limitation that the calculation of ER largely depends on the statistical distribution of the exposures, we further examined the potential proportion declination that would occur if exposure to size-specific particulate matter were reduced to the WHO recommended levels (15 μg/m^3^ for PM_2.5_, 30 μg/m^3^ for PM_c_, and 45 μg/m^3^ for PM_10_). Our counterfactual approach calculated the difference between the observed true number of hospitalizations and the estimated number of hospitalizations in counterfactual scenarios (the WHO recommended levels of air pollutants). Because the concentration of air pollutants for each person was input into the statistical models of the counterfactual analysis, this empirical approach is not subject to the underlying statistical distributions of the air pollutants. Our counterfactual analysis suggested that reducing PM_2.5_ to the WHO reference was associated with the largest potential decline in ALRI outpatient visits, followed closely by the reduction of PM_10_, while reducing PM_c_ to the WHO reference is associated with the lowest potential for a decline in ALRI outpatient visits, which is likely explained by the fact that the mean level of PM_c_ (21.0 μg/m^3^) in our sample is lower than that of the WHO reference level (30 μg/m^3^).

Our counterfactual analysis results have a more practical public health meaning than those of ER. The implication that reducing the level of PM_2.5_ may be associated with the largest decline in ALRI outpatient visits is consistent with the previous studies reporting about the toxicity of smaller-sized particulate matter on lower respiratory infection hospitalizations (36–40). For example, Wang et al. specifically focused on the association between the size-specific particulate matter and childhood pneumonia, and they reported a graded impact of the size-specific particulate matter on the childhood pneumonia (PM_1_ > PM_2.5_ > PM_10_). Smaller-sized particulate matter is more likely to enter the smaller airways and cause severe health consequences.

Although the air quality has been substantially improved attributable to the effort of air quality management in China over the past decade (41, 42). The average level of particulate matter (especially PM_2.5_ and PM_10_) is still above the WHO recommended level. Northern Chinese cities with the high population densities can experience anomalously high levels of air pollution during the winter (43). Our results highlight the importance of focusing on the smaller-sized particulate matter due to its harmful effects on ALRI outpatient visits.

This study should be interpreted in view of several limitations. First, we used daily aggregated data to evaluate the short-term effect of particulate matter on health outcomes, but this aggregated nature of data could be subject to ecological bias. Second, a city-wide average concentrations of air pollution was used to represent the population exposure level, which could lead to exposure misclassification. Third, we included a relatively small number of asthma outpatient visits, which led to unstable point estimates and CIs for asthma. Fourth, since we used secondary data collected from the hospital administrative database, some important confounders (such as maternal smoking, prenatal care, and BMI) were not available to us. Lastly, we only used data from a single hospital, which limits the applicability of the results to the other regions of China.

Nonetheless, this study has several strengths. First, this is the first study to investigate the association between the size-specific particulate matter and subtypes of ALRI outpatient visits, while previous studies either reported the association between PM_2.5_ and subtypes of ALRI outpatient visits or the association between the size-specific particulate matter and overall ALRI hospitalization without details on subtypes (5–7). Second, we used the counterfactual analyses to estimate the potential percent reduction in ALRI outpatient visits compared with the WHO-recommended levels. The results of counterfactual analyses have more substantial public health significance compared with ER, OR, and any other estimates associated with a fixed amount of increase in particulate matter (such as per 10 μg/m^3^ increase in PM_2.5_) (11–13).

## Conclusions

In summary, this study suggests a larger potential percent of the reduction in ALRI outpatient visits if PM_2.5_ could be lowered to the levels recommended by the WHO. The association between particulate matter and pneumonia outpatient visits was stronger among men patients and in the cold seasons. The results highlight the need for a consolidated effort to reduce the particulate matter pollution of smaller sizes and consequently improve the health outcomes of residents in China.

## Data Availability Statement

The data analyzed in this study is subject to the following licenses/restrictions: Ownership of the data does not belong to the individual. Requests to access these datasets should be directed to Chuming You, gd2hek@163.com.

## Author Contributions

ZL: conceptualization, investigation, visualization, writing—original draft, writing—reviewing, and editing. QM: investigation, visualization, and funding acquisition. QY and NC: investigation, writing—reviewing, and editing. CY: investigation, visualization, supervision, and project administration. All authors contributed to the article and approved the submitted version.

## Funding

This study was supported by the Science Foundation of Guangdong Second Provincial General Hospital (2021BSGZ001).

## Conflict of Interest

The authors declare that the research was conducted in the absence of any commercial or financial relationships that could be construed as a potential conflict of interest.

## Publisher's Note

All claims expressed in this article are solely those of the authors and do not necessarily represent those of their affiliated organizations, or those of the publisher, the editors and the reviewers. Any product that may be evaluated in this article, or claim that may be made by its manufacturer, is not guaranteed or endorsed by the publisher.
