# Supplementary material for: Size-Specific Particulate Matter Associated With Acute Lower Respiratory Infection Outpatient Visits in Children: A Counterfactual Analysis in Guangzhou, China
Source: Front Public Health. 2021 Dec 2;9:789542. doi: 10.3389/fpubh.2021.789542 (PMC8674437; doi:10.3389/fpubh.2021.789542)
Supplement: Supplementary file 1 [file Data_Sheet_1.docx]

**Figure S1.** Exposure-response curves for daily concentrations of air pollutants associated with outpatient visits of pneumonia, bronchiolitis, and asthma. Restricted cubic splines with 5 knots were applied to these nonlinear curves.


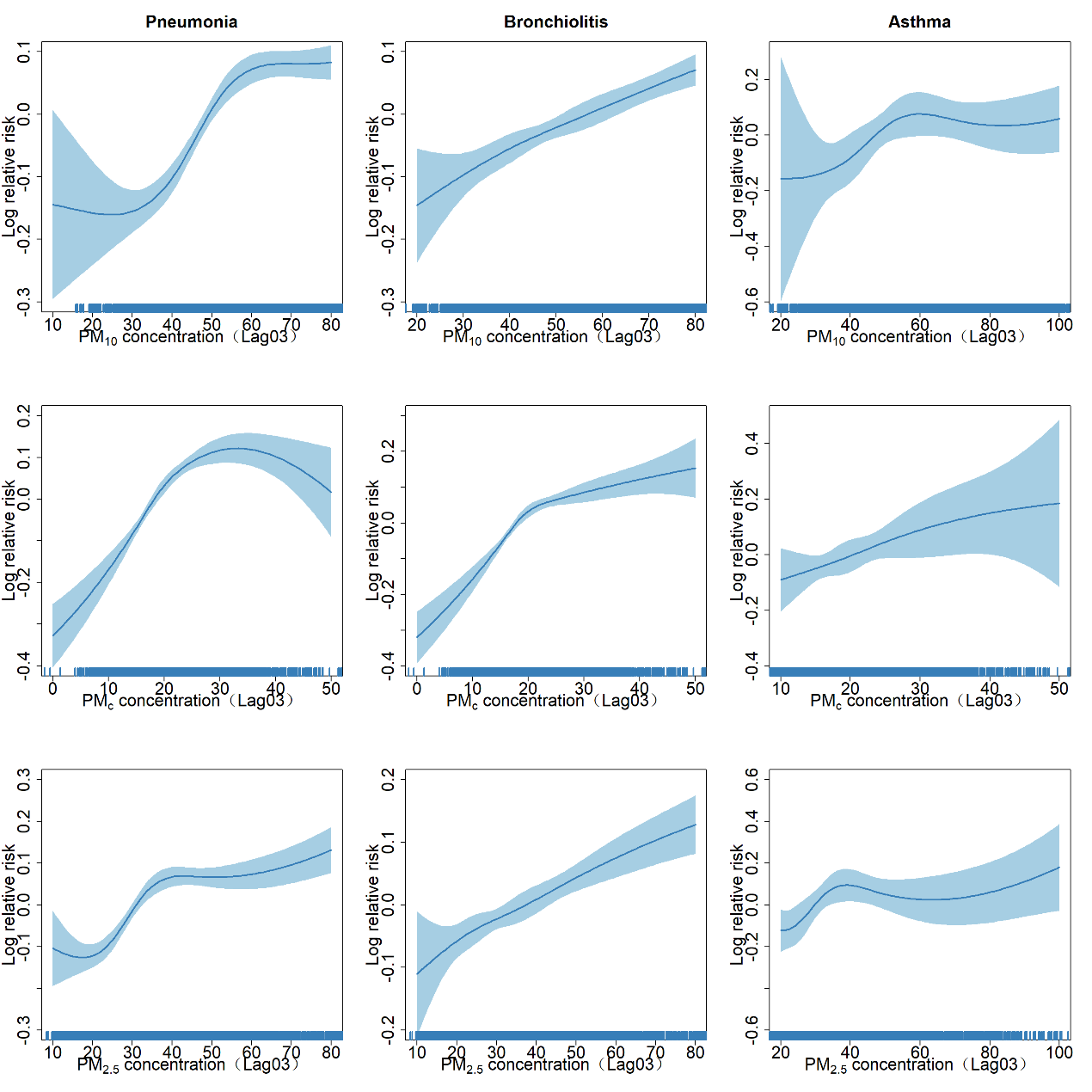


Table S1. Overdispersion test results for generalized linear models for pneumonia, bronchiolitis, and asthma

| Pollutants | Pneumonia | | | Bronchiolitis | | | Asthma | | |
| --- | --- | --- | --- | --- | --- | --- | --- | --- | --- |
|  | Dispersion  estimates | Z-statistic | P-value | Dispersion  estimates | Z-statistic | P-value | Dispersion  estimates | Z-statistic | P-value |
| PM_10_ | 6.481 | 15.126 | <0.001 | 5.375 | 27.117 | <0.001 | 2.089 | 13.340 | <0.001 |
| PM_c_ | 6.672 | 15.026 | <0.001 | 5.355 | 26.906 | <0.001 | 2.184 | 12.584 | <0.001 |
| PM_2.5_ | 6.327 | 15.414 | <0.001 | 5.418 | 27.240 | <0.001 | 2.069 | 13.270 | <0.001 |

**Table S2**. Excess risk and 95% confidence intervals of pneumonia, bronchiolitis, and asthma for each 10 μg/m^3^ increase in PM_2.5_, PM_c_, PM_10_ with different degrees of freedom for splines of temporal trends and temperature.

| Pollutants | Models | Pneumonia | Bronchiolitis | Asthma |
| --- | --- | --- | --- | --- |
| PM_10_ |  |  |  |  |
|  | df=5 for temporal trends | 3.92 (3.10, 4.75) | 3.42 (2.69, 4.17) | 3.65 (1.39, 5.97) |
|  | df=7 for temporal trends | 3.72 (2.90, 4.55) | 3.38 (2.67, 4.10) | 3.47 (1.15, 5.85) |
|  | df=8 for temporal trends | 3.37 (2.56, 4.19) | 3.15 (2.44, 3.86) | 3.29 (1.05, 5.58) |
|  | df=5 for temperature | 3.79 (2.99, 4.60) | 3.29 (2.58, 4.01) | 3.45 (1.18, 5.77) |
|  | df=7 for temperature | 3.67 (2.86, 4.48) | 3.19 (2.47, 3.91) | 3.46 (1.18, 5.78) |
|  | df=8 for temperature | 3.67 (2.87, 4.48) | 3.18 (2.46, 3.90) | 3.46 (1.18, 5.78) |
| PM_c_ |  |  |  |  |
|  | df=5 for temporal trends | 10.86 (8.55, 13.23) | 11.17 (9.06, 13.32) | 10.93 (3.81, 18.54) |
|  | df=7 for temporal trends | 9.56 (7.28, 11.88) | 9.41 (7.40, 11.45) | 11.78 (4.52, 19.54) |
|  | df=8 for temporal trends | 8.58 (6.31, 10.90) | 7.81 (5.83, 9.83) | 8.69 (1.79, 16.06) |
|  | df=5 for temperature | 9.36 (7.12, 11.65) | 9.35 (7.32, 11.41) | 11.69 (4.45, 19.43) |
|  | df=7 for temperature | 9.15 (6.90, 11.45) | 9.12 (7.08, 11.20) | 11.69 (4.46, 19.43) |
|  | df=8 for temperature | 9.14 (6.89, 11.44) | 9.06 (7.02, 11.13) | 11.69 (4.46, 19.42) |
| PM_2.5_ |  |  |  |  |
|  | df=5 for temporal trends | 4.41 (3.23, 5.59) | 3.06 (2.01, 4.12) | 3.44 (0.35, 6.62) |
|  | df=7 for temporal trends | 4.32 (3.15, 5.51) | 3.25 (2.21, 4.30) | 3.12 (-0.02, 6.36) |
|  | df=8 for temporal trends | 4.06 (2.90, 5.24) | 3.55 (2.54, 4.57) | 3.68 (0.61, 6.84) |
|  | df=5 for temperature | 4.47 (3.32, 5.63) | 3.22 (2.20, 4.25) | 3.32 (0.25, 6.48) |
|  | df=7 for temperature | 4.29 (3.13, 5.45) | 3.09 (2.06, 4.12) | 3.33 (0.26, 6.49) |
|  | df=8 for temperature | 4.31 (3.16, 5.48) | 3.10 (2.07, 4.13) | 3.33 (0.26, 6.49) |
